# Effect of post-ruminal guanidinoacetic acid supplementation on creatine synthesis and plasma homocysteine concentrations in cattle

**DOI:** 10.1093/jas/skaa072

**Published:** 2020-03-10

**Authors:** Mehrnaz Ardalan, Erick D Batista, Evan C Titgemeyer

**Affiliations:** 1 Department of Animal Sciences and Industry, Kansas State University, Manhattan, KS; 2 Department of Animal Science, Universidade Federal de Viçosa, Viçosa, Minas Gerais, Brazil

**Keywords:** creatine, guanidinoacetic acid, homocysteine, methionine

## Abstract

Creatine stores high-energy phosphate bonds in muscle, which is critical for muscle activity. In animals, creatine is synthesized in the liver from guanidinoacetic acid (**GAA**) with methylation by *S*-adenosylmethionine. Because methyl groups are used for the conversion of GAA to creatine, methyl group deficiency may occur as a result of GAA supplementation. With this study, the metabolic responses of cattle to post-ruminal supplementation of GAA were evaluated with and without methionine (**Met**) supplementation as a source of methyl groups. Six ruminally cannulated Holstein heifers (520 kg) were used in a split-plot design with treatments arranged as a 2 × 5 factorial. The main plot treatments were 0 or 12 g/d of l-Met arranged in a completely randomized design; three heifers received each main plot treatment throughout the entire experiment. Subplot treatments were 0, 10, 20, 30, and 40 g/d of GAA, with GAA treatments provided in sequence from lowest to highest over five 6-d periods. Treatments were infused continuously to the abomasum. Heifers were limit-fed twice daily a diet consisting of (dry matter basis) 5.3 kg/d rolled corn, 3.6 kg/d alfalfa hay, and 50 g/d trace-mineralized salt. Plasma Met increased (*P* < 0.01) when Met was supplemented, but it was not affected by supplemental GAA. Supplementing GAA linearly increased plasma arginine (% of total amino acids) and plasma concentrations of GAA and creatinine (*P* < 0.001). Plasma creatine was increased at all levels of GAA except when 40 g/d of GAA was supplemented with no Met (GAA-quadratic × Met, *P* = 0.07). Plasma homocysteine was not affected by GAA supplementation when heifers received 12 g/d Met, but it was increased when 30 or 40 g/d of GAA was supplemented without Met (GAA-linear × Met, *P* = 0.003); increases were modest and did not suggest a dangerous hyperhomocysteinemia. Urinary concentrations of GAA and creatine were increased by all levels of GAA when 12 g/d Met was supplemented; increasing GAA supplementation up to 30 g/d without Met increased urinary GAA and creatine concentrations, but 40 g/d GAA did not affect urine concentrations of GAA and creatine when no Met was supplemented. Overall, post-ruminal GAA supplementation increased creatine supply to cattle. A methyl group deficiency, demonstrated by modest increases in plasma homocysteine, became apparent when 30 or 40 g/d of GAA was supplemented, but it was ameliorated by 12 g/d Met.

## Introduction

Guanidinoacetic acid (**GAA**) is a precursor for creatine, which is a compound that allows the storage of high-energy phosphate bonds in muscle (Brosnan et al., 2009; Murakami et al., 2014). Creatine can be supplied through the diet or synthesized endogenously (Brosnan and Brosnan, 2007). Production of GAA occurs via the transfer of an amidino group from arginine to glycine, largely in the kidney (Brosnan and Brosnan, 2004). A methyl group from *S*-adenosylmethionine is transferred to GAA to form creatine, predominantly in the liver; this reaction is catalyzed by guanidinoacetate *N*-methyltransferase. Creatine is released to the blood to be taken up by a tissue such as skeletal muscle. In skeletal muscle, the guanidino group of creatine can accept a phosphate group from adenosine triphosphate to produce adenosine diphosphate and phosphocreatine. Creatinine is a spontaneously produced end product of creatine and creatine phosphate, and it is excreted in the urine (Wyss and Kaddurah-Daouk, 2000).

Methionine (**Met**) is an essential amino acid (**AA**) that is often limiting for cattle (Schwab et al., 2004; Varga, 2010). Activation of Met forms *S*-adenosyl-l-methionine which is the body’s primary methyl group donor. Homocysteinemia can result from conditions where methyl groups are limiting (Brosnan and Brosnan, 2006; Williams and Schalinske, 2007). Because GAA is an obligate consumer of methyl groups, it may produce a methyl group deficiency (Williams and Schalinske, 2010). Some compounds that act as methyl donors, such as Met, choline, and betaine, may prevent methyl group deficiency and the associated homocysteinemia (Tehlivets et al., 2013).

Our hypotheses were that 1) GAA supplementation could create a methyl group deficiency in cattle and 2) Met supplementation could prevent the methyl group deficiency.

## Materials and Methods

All experimental procedures involving cattle were approved by the Institutional Animal Care and Use Committee at Kansas State University.

Six ruminally cannulated Holstein heifers (520 ± 49 kg initial body weight) were used in a 44-d experiment, composed of 14 d for adaptation to facilities and diet and five 6-d periods, with samples collected on days 3 and 6 of each period. The experiment used a split-plot design. The main plot treatments were 0 or 12 g/d of l-Met arranged in a completely randomized design; three heifers received each main plot treatment throughout the entire experiment. Subplot treatments were 0, 10, 20, 30, and 40 g/d of GAA, with treatments provided in sequence from the lowest to the highest level. Treatments were increased over time due to the possibility that the GAA might cause negative health effects on the heifers, and the increasing doses would allow the detection of any problems at the lowest amount that was problematic. In addition, the sequential increase of GAA amounts partially reduced the need for an extended adaptation period because the heifers were adapted to the next greatest amount of GAA prior to initiating each treatment. Treatments were all provided as continuous infusions into the abomasum to preclude the potential for ruminal degradation. Treatments were infused into the abomasum through Tygon tubing (i.d. = 3.32 mm; Saint-Gobain North America, Valley Forge, PA) passed through the ruminal cannula, the reticulo-omasal orifice, and the omasum and held in the abomasum with a circular rubber flange (10 cm diameter) at one end. A peristaltic pump (Model crude protein [**CP**]-78002-10; Cole-Parmer Instrument Company, Vernon Hills, IL) was used to make the infusions.

The treatment solutions of GAA were prepared as 1% solutions in water; the GAA was initially solubilized with 0.22 g of 6 M HCl/g GAA (pH approximately 2.5), and then 0.11 g of 50% (wt/wt) NaOH/g GAA was added, which raised the pH to approximately 3.1. The GAA was then diluted, as necessary, so that the final weight of the solution was 4 kg/d. For those heifers receiving l-Met, it was added to the solution for each heifer and shaken until dissolved.

Heifers were housed in tie-stalls with free access to water and were limit-fed twice daily (0600 and 1800 hours) a diet (Table 1) providing (dry matter [**DM**)] basis) 5.3 kg/d of rolled corn, 3.6 kg/d of alfalfa, and 50 g/d of trace-mineralized salt. Using the NASEM (2016) model, basal supplies of metabolizable Met were estimated to be 17.2 g/d (assumptions: corn = 88% total digestible nutrients [**TDN**], 65% ruminally undegraded CP, 2.1% Met in CP; alfalfa = 60% TDN, 20% ruminally undegraded CP, 1.35% Met in CP). Because the diet contained no animal products, it was assumed devoid of GAA and creatine. Animals were observed daily for any potential symptoms of toxicity such as inappetence or depressed attitude, but no health problems were observed during the study.

**Table 1.** Composition of experimental diet

Ingredient	% of DM
Alfalfa hay^1^	40.31
Dry-rolled corn^2^	59.13
Trace mineral salt^3^	0.56

^1^Composition (DM basis): 36% NDF, 24% CP, 11% ash.

^2^Composition (DM basis): 8% NDF, 9% CP, 2% ash.

^3^Composition (DM basis): > 95.5% NaCl, 0.24% Mn, 0.24% Fe, 0.05% Mg, 0.032% Cu, 0.032% Zn, 0.007% I, and 0.004% Co.

### Sample collection and laboratory analyses

Samples of the feed ingredients (rolled corn and alfalfa) were collected and frozen (−20 °C) on days 3 and 6 of each period for subsequent analysis. Samples were mixed within the period to obtain composite samples, dried in a 55 °C forced-air oven for 72 h to determine partial DM, ground to pass through a 1-mm screen (Thomas-Wiley Laboratory Mill Model 4, Thomas Scientific USA, Swedesboro, NJ), and stored for subsequent analysis. Feed samples were analyzed for DM, neutral detergent fiber (**NDF**), N, and ash. The NDF content was measured according to the technique of Van Soest et al. (1991) with α-amylase and sodium sulfite. DM content was determined by drying samples at 105 °C for 24 h in a forced-air oven. Ash was determined following combustion at 450 °C for 8 h. The N content was measured with a combustion analyzer (Nitrogen Analyzer, Leco Corporation, St. Joseph, MI), and CP was calculated as N × 6.25.

On days 3 and 6 of each period, blood samples were collected from the coccygeal vein into 10-mL heparinized blood collection tubes (Becton, Dickinson and Co., Franklin Lakes, NJ) at 4 h after the morning feeding (1000 hours). Samples were stored on crushed ice immediately after collection and then centrifuged (1,200 × *g*, 4 °C, 15 min) to harvest plasma. Plasma samples were frozen at −20 °C for later analyses of AA (day-6 samples only), homocysteine (day-6 samples only), GAA, creatine, and creatinine. On day 6 of each period, urine samples were collected, following stimulation of the vulva to induce urination, three times at 0700, 1000, and 0100 hours, composited, and frozen at −20 °C for later analyses of GAA, creatine, and creatinine.

Plasma free AA were determined by high-performance liquid chromatography (**HPLC**) after deproteinization by mixing plasma with equal volumes of 10% (wt/vol) sulfosalicylic acid containing norleucine as an internal standard (Campbell et al., 1997). Chromatography was achieved on a Li cation-exchange column, followed by derivatization with *o*-phthalaldehyde and fluorescence detection (Batista et al., 2016).

Plasma homocysteine was analyzed as the carboxymethyl derivative formed as described by Tcherkas and Denisenko (2001) using cation-exchange HPLC separation with post-column *o*-phthalaldehyde derivatization and fluorescence detection. Samples or standards (1 mL) were placed in polyethylene tubes, and disulfide bonds were reduced with the addition of 200 µL 2-mercaptoethanol, which was mixed and allowed to set at 20 °C for 5 min. Samples were then deproteinized with 2 mL of methanol, which was mixed for 30 s. Samples were then centrifuged at 4,000 × g for 15 min at 20 °C. Supernatants (2.25 mL) were then dried in 2-mL microcentrifuge tubes under N_2_, then reconstituted with 50 µL water and 50 µL 2-mercaptoethanol. Carboxymethyl derivatives were produced by the addition of 200 µL of 2 M iodoacetic acid (mixed in 0.1 M sodium borate buffer adjusted to pH 11.5); sample blanks, which were subtracted from concentrations of derivatized samples, were treated with 200 µL of 0.1 M sodium borate buffer adjusted to pH 11.5. Following mixing and a 5-min reaction period, 400 µL of lithium diluent (LI220; Pickering Laboratories, Mountain View, CA) was mixed with each sample, which was subsequently centrifuged at 17,000 × g for 5 min at 20 °C and filtered through a 0.2-µm syringe filter. The HPLC analysis used a cation-exchange column (Pickering Laboratories) maintained at 33 °C, a 20-µL sample loop, and a flow rate of 0.6 mL/min. The eluant was a lithium eluant at pH 2.75 (LI275, Pickering Laboratories) for 20 min, followed by a lithium column regenerant (RG003; Pickering Laboratories) for 5 min, and re-equilibration of the column with LI275 for 20 min prior to the next sample injection. Post-column derivatization of eluted compounds with *o*-phthalaldehyde and 2-(dimethylamino)ethanethiol (both from Pickering Laboratories) was followed by fluorescence detection (excitation 330 nm, emission 465 nm).

Urine and blood GAA, creatine, and creatinine were determined using HPLC according to Shingfield and Offer (1999) with some modification. Plasma samples were prepared by mixing equal volumes of 10% (wt/vol) sulfosalicylic acid and sample, vortexing, freezing overnight, centrifuging (17,000 × *g*, 10 min, 4 °C), and then filtering through a 0.2-µm syringe filter into HPLC vials for injection. For urine sample preparation, 100 µL of sample was diluted with 900 µL diluent, which consisted of 0.9 g of ammonium phosphate and 1.01 g of sodium 1-heptane sulfonic acid in 1 liter of deionized H_2_O with pH adjusted to 2.2 with H_3_PO_4_. Diluted samples were filtered through a 0.2-µm syringe filter into an HPLC vial. The components of the sample were separated on a 25 cm × 4.6 mm Discovery BIO Wide Pore C18 column (5-µm particle size; Supelco Inc., Bellefonte, PA). The mobile phase consisted of 1.01 g sodium 1-heptane sulfonic acid, 0.9 g ammonium phosphate, 35 mL methanol, and 70 µL triethylamine made to 1 L with deionized H_2_O and adjusted to pH 2.8 with 7.5 M H_3_PO_4_. Compounds were detected by absorbance at 200 nm. To achieve chromatographic separation, 5 µL samples were injected to the column at 20 °C with a flow rate of 0.5 mL/min for 14 min, and then with a flow rate of 1.2 mL/min. Sample separation was completed at 25 min, and the column was flushed with 100% methanol for 10 min at 1.2 mL/min and re-equilibrated with the mobile phase for 19 min. The flow rate was then returned to 0.5 mL/min for 1 min prior to the next injection. Total run time was 55 min.

Glomerular filtration was calculated under the assumption that creatinine is entirely filtered from the blood and not reabsorbed from glomerulus. Renal reabsorption of GAA was calculated as: 1 − [(urinary GAA concentration/urinary creatinine concentration)/(plasma GAA concentration/plasma creatinine concentration)], and renal reabsorption of creatine was calculated similarly with concentrations of creatine replacing those of GAA.

### Statistical analyses

Treatments were arranged as a 5 × 2 factorial and included five amounts of GAA (0, 10, 20, 30, and 40 g/d) and two amounts of Met (0 and 12 g/d). Initial analyses were completed with plasma GAA and creatine concentrations to determine if cattle had adapted to treatments by day 3. Data from samples collected on days 3 and 6 of each period were analyzed with a repeated-measures analysis with a model including fixed effects of Met, GAA, day, and all interactions. Heifer within Met level was included as a random effect that served as the error term for testing effects of Met. Day was treated as a repeated measure with a covariance structure of compound symmetry.

Data from day 6 were analyzed with a model including fixed effects of Met, GAA, and their interaction. The GAA supplementation was considered as a repeated measure with the covariance structure of autoregressive. Heifer within Met level was included as a random effect that served as the error term for testing effects of Met. Means were separated using polynomial contrasts to test the linear and quadratic effects of GAA as well as the interactions of Met with those effects.

## Results and Discussion

Creatine is synthesized, predominantly in the liver, from GAA and *S*-adenosylmethionine, which provides the methyl group for synthesis of creatine from GAA. Because regulation of creatine synthesis occurs at the level of GAA synthesis, the use of methyl groups in the conversion of GAA to creatine is an unregulated process (Ostojic, 2014). Thus, GAA supplementation in amounts greater than normal endogenous synthesis can induce a methyl group deficiency and consequently hyperhomocysteinemia (Setoue et al., 2008). Supplemental methyl group sources such as Met, which acts as the methyl donor in most biological methylation, can play an important role in supplying methyl groups to produce creatine. Therefore, providing sufficient methyl groups for GAA methylation not only can increase creatine synthesis but also can prevent the methyl group deficiency which may occur as a result of GAA supplementation (Ostojic, 2014; Peters et al., 2015).

Several studies have elucidated the role of GAA as a creatine precursor for humans and as a means of improving the growth and yield of meat for broilers (Lemme et al., 2011) and growing-finishing pigs (Wang et al., 2012). In contrast, we were unaware of any research with GAA as a creatine precursor for cattle. This work was conducted as a pilot study to provide useful information about GAA utilization and to develop a GAA-induced methyl group deficiency model in cattle. Our goal was to evaluate the ability of post-ruminal supplementation of relatively large amounts of GAA to generate a methyl group deficiency in cattle and to determine if Met supplementation could ameliorate the methyl group deficiency.

### Plasma homocysteine, GAA, creatine, and creatinine concentrations

Data (Table 2) demonstrated significant effects of day as well as interactions of day with treatment: creatine (Met × day, *P* = 0.10; GAA × day, *P* = 0.08; Met × GAA × day, *P* = 0.05), GAA (Met × day, *P* = 0.07; Met × GAA × day, *P* = 0.05). Because the plasma data demonstrated that cattle had not completely adapted to treatments by day 3 of each period, only data from day 6 were used for analyses discussed in this paper.

**Table 2.** Effect of Met and GAA supplementation on plasma concentrations of GAA, creatine, and creatinine on days 3 and 6

Plasma, mg/L	Day		GAA, g/d						*P*-value^1^						
		Met, g/d	0	10	20	30	40	SEM	Day	Met	GAA	M × G	M × D	G × D	M × G × D
GAA	3	0	1.08	0.96	1.57	1.57	2.22	0.25	0.02	0.35	<0.0001	0.32	0.07	0.91	0.05
		12	0.67	1.06	1.19	2.23	2.14								
	6	0	0.98	1.36	2.08	2.63	2.59								
		12	1.19	1.26	1.37	1.85	1.94								
Creatine	3	0	27.1	30.1	32.8	37.8	35.7	2.1	<0.0001	0.51	<0.0001	0.64	0.10	0.08	0.01
		12	26.4	31.0	30.2	33.6	29.1								
	6	0	26.0	31.0	30.9	31.5	25.9								
		12	26.4	28.4	28.0	30.0	29.7								
Creatinine	3	0	9.36	9.73	11.60	10.97	10.96	0.39	<0.01	0.11	<0.0001	0.09	0.32	0.34	0.29
		12	8.49	9.85	9.74	9.89	9.88								
	6	0	9.04	9.57	10.19	9.80	9.99								
		12	8.51	8.78	9.23	9.14	9.59								

^1^M × G = Met × GAA; M × D = Met × day; G × D = GAA × day; M × G × D = Met × GAA × day.

There was a Met × GAA-linear interaction (*P* = 0.003) for plasma homocysteine (Table 3). For heifers receiving no Met, increases in plasma homocysteine were induced by 30 and 40 g/d of GAA, whereas for heifers receiving 12 g/d of Met, there was no increase in plasma homocysteine in response to increasing GAA supplementation. The lack of change in plasma homocysteine concentrations in response to supplemental GAA in the presence of supplemental Met could be a result of the role Met plays in providing enough methyl groups to prevent methyl group deficiency. The increases in plasma homocysteine concentrations in response to GAA infusions in the absence of supplemental Met indicated that a methyl group deficiency was generated, suggesting that consumption of methyl groups for the conversion of GAA to creatine can restrict the availability of methyl groups for other reactions in the body. The methylation of GAA also produces *S*-adenosylhomocysteine, which may contribute to elevated levels of homocysteine in blood (McBreairty et al., 2015). The elevation of plasma homocysteine can be considered a useful marker of methyl group deficiency (da Costa et al., 2005; Setoue et al., 2008). Met prevented the elevation in plasma homocysteine, likely by providing methyl groups that could aid in homocysteine remethylation or by increasing the flux of homocysteine through transsulfuration (Zhou et al., 2017).

**Table 3.** Effect of Met and GAA supplementation on plasma and urinary concentrations of homocysteine, GAA, creatine, and creatinine

		GAA, g/d						*P*-value^1^				
Item	Met, g/d	0	10	20	30	40	SEM	Met	G-L	G-Q	Met × G-L	Met × G-Q
Plasma												
Homocysteine, µM	0	15.4	16.4	16.1	18.5	18.2	1.0	0.27	0.32	0.38	0.003	0.43
	12	16.9	15.6	16.4	13.6	15.8						
GAA, mg/L	0	0.98	1.36	2.08	2.63	2.59	0.24	0.06	<0.001	0.62	0.03	0.26
	12	1.19	1.26	1.37	1.85	1.94						
Creatine, mg/L	0	26.0	31.0	30.9	31.5	25.9	2.4	0.83	0.42	0.03	0.46	0.07
	12	26.4	28.4	28.0	30.0	29.7						
Creatinine, mg/L	0	9.04	9.57	10.19	9.80	9.99	0.32	0.17	<0.001	0.06	0.70	0.12
	12	8.51	8.78	9.23	9.14	9.59						
Urine												
GAA, mg/L	0	67	81	116	106	65	14	0.99	0.06	0.01	0.21	0.06
	12	63	80	94	95	104						
Creatine, mg/L	0	491	618	1,027	829	386	150	0.47	0.35	0.02	0.35	0.03
	12	634	666	861	782	933						
Creatinine, mg/L	0	1,546	1,245	1,597	1,174	1,118	161	0.54	0.02	0.89	0.92	0.56
	12	1,404	1,347	1,334	958	1,172						

^1^G-L, linear effect of GAA; G-Q, quadratic effect of GAA.

Plasma creatine concentrations increased quadratically (*P* = 0.03) with GAA supplementation, with all amounts of supplemental GAA elevating plasma creatine except when 40 g/d of GAA was provided with no supplemental Met. It is possible that methyl group deficiency may have limited the synthesis of creatine from GAA when no Met was provided, preventing cattle from maintaining the higher rates of creatine synthesis when large amounts of GAA were provided.

In our study, supplemental GAA linearly increased (*P* = 0.001) plasma GAA concentrations from 1.08 to 2.26 mg/L, but increases in plasma GAA concentrations tended (*P* = 0.06) to be less when Met was provided, perhaps reflecting that Met produced a more effective uptake of GAA to support creatine synthesis. Some authors have reported increased concentrations of plasma GAA in humans (Ostojic and Vojvodic-Ostojic, 2015) in response to supplementation of GAA. Also, we observed that plasma creatinine concentrations increased linearly (*P* = 0.001) with GAA administration. Because of the conversion of creatine to creatinine, it was not surprising that plasma creatinine was elevated by GAA supplementation.

Ostojic et al. (2013b) studied 20 healthy human volunteers who consumed GAA with and without methyl group donors. Total plasma homocysteine was increased significantly by oral administration of GAA alone, whereas there was no significant difference in total plasma homocysteine when GAA was supplemented along with methyl donors. Additional work was conducted by Ostojic et al. (2014b) with 48 healthy volunteers consuming three different dosages of GAA (1.2, 2.4, and 4.8 g/d) for 6 wk to determine the effects of GAA supplementation on serum and urinary metabolites. They observed plasma homocysteine increased by 1.4 (15%), 2.6 (30%), and 6.6 μM (78%) when low, medium, and high levels of GAA were provided. In addition, the authors observed that serum GAA, creatine, and creatinine increased substantially in response to GAA supplementation. These results show that increasing exogenous GAA intake can increase serum GAA and creatine. This response would be consistent with increased creatine synthesis followed by transport via the bloodstream to various tissues, such as muscle (Ostojic et al., 2014b).

McBreairty et al. (2015) studied the effects of GAA and creatine loading on tissue creatine stores for 18 to 19 d in Yucatan miniature pigs, and they found that hepatic creatine concentrations were significantly greater for creatine and GAA groups compared with the control group. In that study, GAA supplementation led to approximately 2-fold greater hepatic creatine concentrations than did creatine supplementation. Furthermore, supplementation of GAA increased muscle creatine more (~20%) than did creatine supplementation, and it significantly increased plasma creatine concentrations (~70%) compared with the control group. Also, GAA supplementation improved tissue creatine concentration in male broiler chicks (Lemme et al., 2007; Michiels et al., 2012). Murakami et al. (2014) studied quail fed corn–soybean meal basal diets with different dietary levels of GAA (0.00%, 0.06%, 0.12%, 0.18%, and 0.24%), and they observed elevated GAA, creatine, and creatinine content of eggs with increasing dietary levels of GAA, suggesting that GAA supplementation increased GAA conversion to creatine, with increased creatine transfer into the egg.

### Urinary concentrations of GAA, creatine, and creatinine

The body needs creatine for muscle mass development. The requirement for creatine is greater for growing animals than for adults because of the need for creatine to support growing tissues and to replace creatine lost as creatinine (Brosnan et al., 2009; Brosnan and Brosnan, 2010). Because there is a limited capacity to store creatine in tissues, muscle cannot be overloaded by circulating creatine (DeGroot et al., 2018).

In our study, supplemental GAA (Table 3) quadratically elevated (initial increase followed by plateau; *P* = 0.03) urinary concentrations of GAA. The shapes of the curves tended to be affected by Met supplementation (Met × GAA-quadratic; *P* = 0.06) because heifers receiving no supplemental Met demonstrated a decrease in urinary GAA concentrations when 40 g/d GAA was supplemented, but heifers receiving 12 g/d Met did not. Considering an estimated urine output of 10 L/d, urinary GAA excretion was increased by not more than 0.4 g/d, even when the highest amount of GAA was supplemented, demonstrating that the preponderance of supplemental GAA was methylated to creatine.

Urinary concentrations of creatine increased quadratically (*P* = 0.02) in response to GAA supplementation in parallel with increases in plasma creatine. Like plasma creatine, when 40 g/d of GAA was provided without supplemental Met, there was a decrease in urinary creatine concentrations, but this decrease was not observed when 12 g/d Met was supplemented (Met × GAA-quadratic; *P* = 0.03). The increases in urinary creatine concentrations in response to GAA supplementation were associated with elevated plasma creatine concentrations, which likely resulted from increased creatine synthesis. When the body is faced with increased GAA supply, much of the GAA is methylated to creatine, but the excess creatine will be eliminated through renal excretion which is the major clearance mechanism for the body (Ostojic et al., 2014a). Similar to our results, Ostojic et al. (2014b) observed progressive increases in serum and urinary concentrations of GAA and creatine when increasing dosages of GAA (1.2, 2.4, and 4.8 g/d) were consumed by healthy humans for 6 wk.

Urinary creatinine concentration linearly decreased (*P* = 0.02) with GAA supplementation. The reason for the reduction of urinary creatinine in response to increasing amounts of GAA is unclear, but it is possible that increases in urine volume could be responsible. Increases in urine volume, however, probably could not account for the decrease in urinary creatine concentration in heifers receiving 40 g/d GAA without Met because the magnitude of drop was not as great for creatinine as for creatine.

There was a Met × GAA-linear interaction for renal reabsorption of GAA (Table 4) because renal reabsorption percentage increased with GAA supplementation when no Met was supplemented, but it decreased with GAA supplementation when 12 g/d Met was supplemented. Supplemental GAA linearly (*P* < 0.01) and quadratically (*P* < 0.01) decreased creatine reabsorption. We also observed a significant interaction between Met and GAA for creatine reabsorption (Met × GAA-quadratic; *P* = 0.01) because heifers receiving 40 g/d GAA without Met demonstrated much greater reabsorption than those that received 12 g/d Met along with 40 g/d GAA. Renal reabsorption of creatine might be expected to decrease as the availability of creatine increases and the body retains less of the available creatine. Although a portion of the changes in urinary creatine concentrations in response to GAA provision can be attributed to changes in plasma concentrations, differences in renal reabsorption were more quantitatively important in affecting urinary creatine concentrations.

**Table 4.** Effect of Met and GAA supplementation on renal reabsorption of GAA and creatine

Metabolite	Met, g/d	GAA, g/d						*P*-value^1^				
		0	10	20	30	40	SEM	Met	G-L	G-Q	Met × G-L	Met × G-Q
		% renal reabsorption										
GAA	0	56.8	53.6	64.7	65.9	77.7	5.6	0.05	0.47	0.11	<0.001	0.77
	12	67.2	58.7	50.2	51.1	52.1						
Creatine	0	90.2	85.2	78.0	77.7	88.6	2.5	0.10	<0.01	<0.01	0.13	0.01
	12	84.0	84.8	77.8	75.2	74.6						

^1^G-L, linear effect of GAA; G-Q, quadratic effect of GAA.

Heifers excreted significant amounts of creatine in the urine, even when no GAA was supplemented. Assuming urine outputs of 10 L/d, control heifers excreted about 5 g/d of creatine, which would seem to be an insensible loss of both N and methyl groups. Previous work has shown that cattle (Dinning et al., 1949), as well as other species (Joncquel-Chevalier Curt et al., 2013), excrete significant amounts of creatine, although researchers have focused more on the urinary losses of creatinine. Thus, our work, in agreement with previous observations, suggests that urinary losses of both creatine and creatinine might be of significant biological and nutritional significance, particularly with regard to requirements for methyl groups as well as for endogenous losses of N. Across our treatments, urinary concentrations of creatine ranged from 32% to 82% of creatinine. Despite statistically significant changes in urinary GAA concentrations, the amount of GAA lost in the urine was not very important quantitatively in comparison to creatinine and creatine; urinary GAA excretion ranged from 3% to 5% of creatine plus creatinine losses.

As a whole, there were notable increases in plasma and urinary concentrations of both GAA and creatine as increasing amounts of GAA were supplemented. However, two of the three heifers receiving 40 g/d of GAA without supplemental Met demonstrated marked decreases in urinary concentrations of GAA and creatine; urinary creatinine was also lower for those two heifers, but the magnitude of drop was not large. Only one of those two heifers demonstrating low urinary GAA and creatine concentrations had a low plasma creatine concentration and neither had strikingly low plasma GAA. Although we did not observe any evidence of health problems in any of the heifers, it is possible that the unusual plasma and urinary concentrations of GAA and creatine could have been a precursor to problems that could have developed if the treatment had been extended for more than 6 d. As noted above, the treatment providing 40 g/d GAA without Met was characterized as having greater renal absorption than would have been predicted from the responses to GAA supplementation up to 30 g/d, demonstrating that the urinary concentrations of GAA and creatine could not be fully explained by changes in plasma concentrations, particularly for GAA. This suggests that some metabolic effects downstream of creatine synthesis may have been affected by the methyl group deficiency noted for that treatment. A precise explanation for the metabolic changes in those two heifers remains unclear.

### Plasma amino acids

The effect of GAA and Met supplementation on plasma AA (% of total) is shown in Table 5. We report these values as percentages of total AA because GAA supplementation quadratically increased total AA concentration (*P* = 0.01; Table 5) and expression as a percentage of the total removes some of the variation associated with individual AA concentrations.

**Table 5.** Effect of Met and GAA supplementation on plasma AA concentrations

		GAA, g/d						*P*-value^1^				
AA	Met, g/d	0	10	20	30	40	SEM	Met	G-L	G-Q	Met × G-L	Met × G-Q
Total AA, mM	0	2.03	2.21	2.37	1.91	2.11	0.11	0.65	0.96	0.01	0.67	0.43
	12	1.93	2.12	2.26	2.15	1.96						
		% of total AA										
Methionine	0	1.13	1.12	1.23	1.22	1.15	0.10	<0.001	0.15	0.36	0.40	0.98
	12	1.72	1.61	1.77	2.06	1.76						
Taurine	0	1.47	1.51	1.31	1.66	1.53	0.16	0.07	0.38	0.74	0.10	0.42
	12	1.79	2.00	1.84	1.55	1.58						
Arginine	0	5.13	5.79	5.85	5.53	6.11	0.26	0.64	0.002	0.11	0.73	0.51
	12	4.88	5.99	5.65	5.44	5.86						
Ornithine	0	3.11	3.60	3.49	2.96	3.30	0.16	0.08	0.28	0.03	0.75	0.49
	12	2.92	3.33	3.34	2.91	2.89						
Citrulline	0	3.62	3.89	3.81	3.53	3.71	0.31	0.40	0.78	0.31	0.97	0.69
	12	3.16	3.74	3.55	3.13	3.39						
Leucine	0	7.38	6.98	7.67	7.17	6.53	0.47	0.24	0.50	0.28	0.53	0.56
	12	6.76	5.89	6.42	7.33	6.02						
Isoleucine	0	6.25	5.44	6.31	5.86	5.52	0.44	0.16	0.43	0.58	0.87	0.36
	12	5.85	4.62	4.73	5.88	4.87						
Valine	0	10.93	10.85	11.41	11.58	10.89	0.60	0.05	0.70	0.69	0.96	0.50
	12	10.02	9.14	9.65	10.42	9.63						
Lysine	0	4.37	4.43	4.32	4.13	4.64	0.37	0.55	0.53	0.59	0.78	0.14
	12	4.20	4.69	4.81	4.77	4.47						
Histidine	0	3.82	3.89	3.45	3.84	3.30	0.20	0.35	0.05	0.11	0.81	0.47
	12	3.80	4.24	3.97	3.71	3.63						
Threonine	0	4.00	4.37	4.72	4.39	4.36	0.19	0.86	0.05	0.22	0.86	0.06
	12	4.26	4.12	4.28	4.52	4.50						
Tryptophan	0	1.41	1.50	1.68	1.73	1.95	0.08	0.30	<0.001	0.68	0.01	0.34
	12	1.47	1.64	1.63	1.58	1.63						
Phenylalanine	0	2.51	2.34	2.87	2.71	2.73	0.16	0.22	0.07	0.30	0.79	0.96
	12	2.83	2.65	2.89	3.23	2.84						
Tyrosine	0	2.49	2.45	3.25	2.54	3.06	0.22	0.60	0.006	0.36	0.78	0.07
	12	2.88	2.44	2.75	3.15	3.25						
Glutamate	0	3.68	3.26	3.02	3.36	3.63	0.27	0.17	0.69	0.003	0.69	0.79
	12	4.17	3.80	3.58	3.41	4.16						
Glutamine	0	15.89	14.16	13.46	14.87	15.02	1.17	0.81	0.89	0.07	0.60	0.40
	12	13.89	14.92	13.90	13.65	15.42						
Aspartic acid	0	0.62	0.56	0.47	0.49	0.52	0.05	0.39	0.001	0.01	0.55	0.99
	12	0.69	0.60	0.54	0.51	0.55						
Asparagine	0	1.68	1.73	2.04	1.66	1.84	0.10	0.95	0.001	0.98	0.03	0.87
	12	1.60	1.63	1.80	2.06	1.87						
Alanine	0	8.53	8.91	8.47	8.52	8.64	0.37	0.01	0.08	0.08	0.11	0.08
	12	11.05	9.92	9.60	9.22	9.81						
Serine	0	3.80	3.74	3.71	3.48	3.57	0.20	0.24	0.24	0.06	0.03	0.03
	12	3.81	4.03	4.08	4.26	3.82						
Glycine	0	8.00	9.37	7.39	8.63	7.92	0.48	0.92	0.17	0.14	0.58	0.57
	12	8.10	8.84	9.08	7.10	7.94						

^1^G-L, linear effect of GAA; G-Q, Quadratic effect of GAA.

In our study, supplemental Met significantly increased plasma Met (*P* < 0.001), but GAA supplementation had no effect on plasma concentrations of Met, which suggests that the methyl group deficiency observed with the higher amounts of GAA was not severe enough to substantially disrupt Met metabolism. Because the synthesis of creatine from GAA requires a methyl group from *S*-adenosylmethionine, we hypothesized that GAA supplementation might decrease Met availability to the heifers either by increasing Met consumption for this process or by increasing transsulfuration. The lack of effect of GAA on plasma Met concentrations might suggest that either Met consumption was not markedly increased by GAA provision or the heifers were able to compensate by remethylating homocysteine and preventing a net consumption of Met. Plasma taurine, a product of sulfur AA metabolism (Stipanuk and Ueki, 2011), tended to be greater (*P* = 0.07) when Met was supplemented. Plasma valine (*P* < 0.05) was reduced by supplementation of Met, an effect that has been attributed in Met-deficiency models to increases in protein deposition (Campbell et al., 1996); it is unknown if our heifers were Met deficient.

The synthesis of GAA occurs through the conversion of arginine and glycine to GAA (Ostojic et al., 2013a) by arginine:glycine amidinotransferase enzymatic activity in the kidney (Brosnan and Brosnan, 2004). The supply of GAA in an animal is normally limited by the renal synthesis of GAA, which is feedback regulated such that GAA production matches the need for creatine (Stead et al., 2001). This regulation prevents the wasteful production of excess creatine. Hepatic methylation of GAA, however, does not appear to be regulated, so all GAA is methylated to form creatine (McBreairty et al., 2013). A reduction in arginine:glycine amidinotransferase activity was found in rat kidney following an increase in serum concentrations of creatine, demonstrating the inhibitory effects of creatine on arginine:glycine amidinotransferase (McBreairty et al., 2013); therefore, the availability of creatine plays a role in the regulation of renal GAA production (Edison et al., 2007). Overall, GAA supplementation in amounts exceeding renal production can reduce renal GAA synthesis, can lead to an obligatory methylation reaction that would irrevocably consume methyl groups, and can have a sparing effect on arginine (Ostojic, 2015). When Dilger et al. (2013) fed young chicks arginine-deficient diets, 0.12% supplemental GAA improved growth responses compared with 0.15% creatine and 0.25% arginine supplementation, indicating that dietary arginine could be replaced by GAA for young chicks. This is consistent with our results in that plasma arginine was increased (*P* = 0.002) by GAA supplementation, particularly with the initial dose of GAA. Increases in plasma arginine in response to supplemental GAA are likely the result of an arginine-sparing effect of GAA supplementation, likely by inhibiting GAA synthesis in the kidney. Quadratic effects were observed for plasma ornithine (*P* = 0.03) and glutamate (*P* = 0.003) for heifers receiving GAA supplementation. The effect of GAA supplementation on plasma ornithine may relate to increases in arginine availability, which could cause more ornithine to be formed from arginine.

Plasma tryptophan, tyrosine, threonine, and asparagine linearly increased (*P* ≤ 0.05) with GAA supplementation. In addition, histidine was linearly decreased (*P* = 0.05) and aspartic acid was linearly and quadratically decreased (*P* ≤ 0.01) by supplemental GAA. The causes and consequences of these changes are unknown.

## Conclusions

The results of this experiment demonstrate that post-ruminal GAA supplementation provides a way to increase creatine supply to cattle. In our study, GAA was provided through post-ruminal infusions, which precluded ruminal microbial degradation of the GAA; the potential for ruminal degradation would need to be considered if GAA were provided through the diet. For large amounts of GAA supplementation (38.5 mg/kg body weight daily), there might be a concern about a methyl group deficiency in response to GAA, although this could be ameliorated by supplemental Met. Methyl group sources other than Met may also ensure against this problem, although we did not evaluate any methyl group sources besides Met. In addition to developing a useful model to assess methyl group utilization in cattle, we were also able to demonstrate for the first time that GAA is used by cattle as a precursor for synthesizing creatine. This research has direct implications for both the dairy and beef cattle industries, because GAA may be an economical means of improving creatine status of cattle.

## Abbreviations

AA: amino acid
CP: crude protein
DM: dry matter
GAA: guanidinoacetic acid
HPLC: high-performance liquid chromatography
Met: methionine
NDF: neutral detergent fiber
TDN: total digestible nutrients
